# Actinomycosis about the Mouth

**Published:** 1900-08

**Authors:** Charles A. Porter

**Affiliations:** Boston, Mass.


### ACTINOMYCOSIS ABOUT THE MOUTH.[1]

BY CHARLES A. PORTER, M.D., BOSTON, MASS.

Mr. President and Gentlemen,—I should not appear again within the year at one of your meetings if it did not seem to me that the subject of this paper was of great interest to you. In years gone by the dentist used to transfer alveolar abscesses, and similar processes about the mouth, to the surgeon for operation; now much oral surgery very properly comes into the province of the dental surgeon, and from our general hospitals more cases are referred to you every year for diseases of the jaw.

Though actinomycosis has been considered a relatively rare disease, it seems more probable that it is one which is very commonly overlooked. In its clinical aspects there is little that is characteristic. Though the course of the infection may make the surgeon suspicious, examination by microscope and culture is essential for a positive diagnosis.

The object of this paper is to attract your attention to the possibility that a proportion of the cases ranking as alveolar abscesses may be due to this specific organism, and by a few briefly reported cases to give you an idea of the disease as it affects the mouth, jaws, and neighboring regions in man. Statistics of the relative frequency of this disease are really of little value. In 1892 Illich, of Vienna, gathered only forty-two cases in man from all that at that time were reported. Dr. Rührah, of Baltimore, has published an article in the *Annals of Surgery,* 1899, in which he has collected all the American cases, seventy-two in number, occurring in all parts of the body. In the past two years I have been especially interested in this subject, and during eighteen months' work in the out-patient department at the Massachusetts General Hospital I have found eight cases of actinomycosis in

---

[1] Read before the American Academy of Dental Science, Boston, May 2, 1900.

about sixty so-called alveolar abscesses examined, and four cases have been found by other surgeons. Six cases of mine have occurred within three months, and four within one week; so we may conclude, I think, that the disease cannot be one of great rarity.

I will pass about some photographs of these cases, as well as a painting of the pus from the abscesses, which shows very well the gray or grayish-yellow granules which may be found in typical cases.

I. Joseph Craven, aged thirty-two; residence, Brookline. Coachman. Entered out-patient department November 7, 1899.

Five weeks ago noticed a small lump on inner side of right lower lip; this grew larger, without marked pain, until two weeks ago, when an inflamed area was evident on the outside of the lip. Examination showed a red inflamed nodule, size of a five-cent piece, on right lower lip; the surrounding tissue was indurated to the size of a quarter; fluctuation was evident in the centre; no communication with mouth. Under cocaine a small incision was made on the inside of the lip; about half a teaspoonful of sero-pus escaped, with numerous granules, which showed the typical appearance of actinomycosis under the microscope. Simple excision was advised, but the patient would consent only to a thorough curetting through an external opening; the surface was disinfected with peroxide, painted with iodine, and packed with iodoform gauze. In two weeks the small wound had almost closed with considerable induration at the base. No colonies could be found in the discharge.

December 15, five weeks after operation, only a soft red scar remained; no induration. Whether this infection started from the inside of the mouth cannot be determined; there were no carious teeth, nor any history of a wound. Many sections of the tissue were examined, but no colonies could be found.

II. William Cowan, aged forty-five; lives in Lowell, Mass. Entered out-patient department August 29, 1899.

Two months ago stuck a toothpick under his tongue, and could not remove the whole of it. One week after this the submaxillary region began to swell. The swelling was varied in amount; no pain; tenderness slight; no interference with talking, eating, or swallowing.

Examination: In the submaxillary region on the left side is an indefinite swelling about the size of a lemon; the skin is somewhat œdematous; at the bottom of the swelling is a small area where fluctuation can be made out; over this the skin is slightly reddened. There are no glands in the neck. Under cocaine a small incision was made. Sero-pus escaped, with a few grayish granules, which proved to be actinomyces.

On the following day a more extensive operation was done under ether. Incisions were made about the involved skin, and the whole submaxillary region thoroughly cleaned from below upward. The submaxillary and a few lymphatic glands were removed with the mass. Just under the jaw a dense fibrous cord was found, extending upward to the floor of the mouth. Within this connective-tissue tube lay the remains of the toothpick lost two months before. This was removed, the flaps loosely sutured, and an iodoform wick placed to the top of the wound. Under daily packings and iodine the wound healed solidly in five weeks, without evidence of recurrence. This case shows well the common connection of actinomycosis with some foreign body.

III. Fred. Legg, aged sixteen, school-boy; residence, Cambridge. Entered out-patient department November 9, 1899.

Teeth have always been bad; has had five removed from upper jaw. Second left molar decayed for some time; two months ago a small lump appeared inside mouth about root of this tooth; this gradually grew in size, without pain, and appeared on the outside of the jaw ten days ago as a reddened semi-fluctuant swelling the size of a quarter, surrounded by a hard and firm border. Inside the mouth a distinct induration could be detected, as of a connective-tissue sinus leading from the tooth to the external swelling. There was moderate trismus; no pain; tenderness slight; no glandular enlargement. A small incision revealed several granules of actinomycosis. Under ether the edges of this wound were excised and the walls of the cavity cut away with scissors; the base thoroughly curetted and painted with iodine. No sinus could be found connecting with the tooth. The wound granulated slowly, but was healed by December 7, when there appeared on the outer side of the scar a small fluctuating area. This was opened and curetted; in the pus no colonies could be found after very careful search.

*December 20.*—The induration within the mouth still persisted, but by January 2 this too had disappeared, and there was then no sign of recurrence.

At operation many granules were obtained which varied in size from a pin-head to three times that size. There was considerable soft, cheesy-looking material in the wall of the cavity. Pure cultures from this case were finally obtained by Dr. Wright.

IV. William Warren, aged thirty, West Everett; teamster. Entered out-patient department November 13, 1899.

Five months ago noticed lump inside mouth opposite last molar teeth on right. This grew larger, and becoming very painful, he went to Emergency Hospital, where it was lanced, with immediate relief. In another month abscess reformed and face swelled to the eye. After lancing no further trouble until three weeks ago, when he noticed "pimple" on outside of jaw about the middle of horizontal ramus; this grew larger, with much swelling and pain.

On entrance the whole right side of the face and eye were much swollen; trismus was well marked; over the centre of the jaw was a reddened, fluctuating lump the size of an English walnut. The surrounding tissues were very hard and brawny. This induration ended very abruptly and gave place to general œdema; within the mouth is well-marked induration from first molar to wisdom-tooth.

Under cocaine the external abscess was opened and *very* numerous granules, a hundred or more, poured out, in thin sero-pus. Some of these were unusually large, almost the size of a very small split pea.

Under ether the infected skin was freely removed, with the base of the cavity down to sound muscular tissue. Again no sinus connecting with the teeth could be found. Closer to the jaw a small cavity was found containing thick, yellow, stinking pus. The wound was dressed as before.

Within three days the swelling had much diminished and the trismus was much less.

December 10, almost a month after the operation, the face suddenly began to swell again. Under ether an abscess to the outer side of the upper jaw was evacuated within the mouth; the pus was foul and contained many soft, yellow granules, appearing somewhat like actinomycosis, but under the microscope proved to be masses of mouth bacteria and leptothrix buccalis.

Just back of the scar of the first operation a "crater" was found, which when opened allowed the little finger to enter nearly to the lower jaw outside of the last molar; the sinus was lined with very dense connective tissue and contained foul pus. Here one or two disorganized colonies of actinomycosis were found.

*January 5.*—In three weeks the swelling and most of the induration had gone; the sinus was healed, and the patient in much better condition.

*March 20.*—Patient reported; no sign of recurrence; no induration; mouth opens normally; about the wisdom-tooth there is some exuberant gum.

In this case the focus of the disease was probably not reached until the last operation, and mixed infection undoubtedly played an important *rôle.* Pure cultures were finally obtained by Dr. Wright from the granules in spite of the contamination.

V. Herbert Grirow, aged twenty-two, 1113 Harrison Avenue.

*October 10, 1899.*—Five weeks ago, without toothache or external wound, a small swelling formed below the middle of the right lower jaw; there was little pain. It has grown considerably in size within the past few days. The skin is hardly reddened over it, but fluctuation is evident. From the upper part of the tumor a firm cord runs upward for one-half an inch; the whole mass is movable and seems rather superficial. Temperature 101° F.; pulse not elevated; teeth not carious. Incision showed the streptothrix colonies. Under ether the diseased skin was excised and the whole mass removed, with a small portion of the submaxillary gland which was adherent to it. No trace of sinus leading to mouth could be made out. The excised mass showed a central cavity surrounded by dense tissue, here and there infiltrated by small areas of grayish-red granulating tissue, containing the pearly granules; some of these show slightly darker centres. In the contents of the cavity were several black bodies, which under the microscope were seen to belong to some beetle. Whether this entered through the floor of the mouth and gradually worked down, or came from the tonsil or œsophagus, is not clear. A man could hardly get the body of a beetle into a wound of the face without knowing it. No history of any external wound could be obtained. Evidently the fungus entered with the beetle. The wound was soundly healed in two weeks.

VI. Mike Clarke, aged twenty, laborer, Cambridgeport. Entered out-patient department November 14, 1899.

Three years ago patient had similar lump in the same place, which broke and went away. This swelling began a week ago as a little round, painless lump.

Below horizontal ramus of right jaw is an oblong red swelling size of peanut, which fluctuates in the centre. From this to the level of the lip the tissue is firm, swollen, and boggy. There is also some swelling over and about the submaxillary gland. Trismus is well marked; can barely open mouth one-fourth inch. On opening the small superficial abscess, a thin, sero-purulent discharge appeared with several colonies; at a deeper level, three drachms foul pus was found, without actinomyces colonies. Apparently diseased tissue; curetted thoroughly, and cavity drained after painting with iodine. In a week the œdema had disappeared and the trismus was much less. Examination of the mouth showed that the three right molars were all carious. In three weeks wound soundly healed; normal motion of jaw.

*March 18.*—Two months afterwards another abscess rapidly formed, which Dr. Balch opened, and Dr. Wright found to contain two typical granules.

March 25 Dr. Balch kindly asked me to see the case again. Trismus was well marked. At the site of the old scar is a small sinus from which a little bloody serum comes. The scar is dense and firmly adherent to the anterior edge of the masseter. Under the jaw are two enlarged glands. Under ether I excised the mouth of the fistula with the surrounding skin. The wall of this sinus was surrounded by very dense connective tissue, which ended abruptly at the cavity, which was lined with soft, flabby granulations. Following along this sinus an inner cavity was found, the inner wall of which was formed by the periosteum of the jaw. From this another small sinus could be traced backward for an inch and a half to the base of the second molar tooth. The whole sinus and cavity was excised and the three carious teeth removed; iodine and gauze drainage; few stitches.

In the centre of the second molar tooth a fairly characteristic granule was found, but on microscopic examination this proved to be a mass of mouth bacteria. The sections have not yet been examined. The case is now entirely healed.

VII. John Smith.

Six months ago broke left lower jaw just anterior to masseter muscle. The bone healed, but whenever he got drunk he had soreness and swelling at the point of fracture. Once a little pus was discharged into his mouth. He came to hospital with a small fluctuating abscess just under the skin. On opening this the typical granules were found. The abscess cavity was thoroughly excised; no bare bone was found. In two weeks the wound was healed. There has been no recurrence. The natural diagnosis was necrosis after fracture; yet no dead bone was found, and thorough excision stopped the spread of the disease.

VIII. Patrick Sharkey, aged forty (Dr. G. W. W. Brewster's case).

For two years has had trouble with left molar teeth, which are carious. Has had several small abscesses opened inside the mouth. In January left side of face swelled and large abscess was evacuated. Since then there has been intermittent purulent discharge into the mouth, and jaw has been sore.

Patient entered on April 15. The left side of the face was much swollen; he could hardly open the mouth; in front of the masseter muscle, over the horizontal ramus of the jaw was a reddened fluctuating abscess the size of a walnut. This was opened and found to contain the granules.

Under ether the abscess cavity was dissected out, and a sinus found leading back to the second molar tooth; here a small piece of dead bone was found. The sinus passed between the jaw and the masseter muscle, which was also involved in the disease. The whole cavity was thoroughly curetted and cauterized, the bad tooth extracted. In ten days the patient had only a small sinus left, which was granulating in a healthy manner.

This case is a good example of the recurrent abscesses which occur until the disease has been thoroughly removed.

The infection seems to enter most frequently near a carious tooth, or is carried in by a foreign body through the mucous membrane of the mouth or pharynx. The process is essentially subacute or chronic, and the disease tends to advance by a sinus towards the skin. Infection is rarely pure, but is usually mixed with ordinary pyogenic organisms or mouth bacteria. It is rarely painful, and the accompanying pain, when it occurs, is due, I think, to the mixed infection. Clinically and under the microscope the disease is characterized by the formation of an unusual amount of dense connective tissue, which ends more or less abruptly at the periphery and infiltrates the adjacent muscle or fat. In the jaw the bone itself is rarely involved, in human actinomycosis, though it may be thickened from periostitis.

It would seem that this surrounding connective tissue could later become infiltrated by the growth of the streptothrix and break down. In all the cases I have examined the inner wall of the cavity shows a clearly cut line of demarcation between the connective-tissue wall and the lining flabby, soft, grayish-red, granulating tissue. Glandular enlargement is conspicuous in its absence, and when present seems to be due to mixed infection. Metastasis seems to occur through the blood-current and not by way of the lymphatics. In serious cases the disease may progress down the neck, into the antrum or through the base of the skull.

Though a definite connection cannot be always demonstrated, it would seem that a sinus at one time leads from the original site to the superficial abscess. In Clarke's case, for example, at the last operation such a sinus was found leading directly to the carious tooth.

Trismus, though often present, is no more characteristic of this disease than of other inflammatory affections, though if the masseter were involved in the dense connective tissue, the jaw would probably remain stiff for a long time.

It is rarely possible, I think, to make a clinical diagnosis of actinomycosis, recurrent abscesses, without necrosis; chronic, painless, subcutaneous abscesses about the jaw, evidently not connected with tubercular glands, would lead to a suspicion of this disease. If these fluctuating areas were surrounded by especially firm and hard connective tissue, and a sinus could be felt under the skin, if there was little œdema and swelling, perhaps a probable diagnosis could be made.

Examination of the discharge is of great assistance, but the mere presence of the so-called "sulphur granules" is not by any means conclusive, and no case should be considered as one of actinomycosis without competent microscopic examination. Small round masses of fibrin or tubercular *débris* sometimes stimulate a colony, in the mouth or adjacent regions. Round masses of mouth bacteria, or leptothrix buccalis, occasionally appear very like a true colony. Even under low powers the resemblance is very similar. Dr. Wright has kindly photographed for me one of these masses, removed from Clarke's tooth. It presents a radiating arrangement, but under a higher power is seen to consist of vast masses of bacilli and the large, thick, non-branching filaments of the leptothrix buccalis.

In examining for actinomycosis gauze sponges which absorb the discharge should not be used. All bleeding, when possible, should be stopped before opening the abscess-wall. Unless badly contaminated actinomycosis pus appears usually as a clear, perhaps blood-tinged, slightly syrupy, sero-pus. Placed on a cover-glass, the granules vary in size from a millet-seed to the head of a large pin. They are usually round, with a clear-cut periphery; the color is gray or grayish-yellow, often suggesting a small pearl; the centre is not rarely somewhat darker. The surrounding pus is non-adherent and the granules can be readily removed alone. Fluid should be examined *at once,* for these granules are found with great difficulty when the blood has once clotted.

With reference to treatment, two facts speak strongly, I think, for the self-limitation of the disease in the majority of cases.

1. Though it cannot be a rare affection, few cases enter the hospital with advanced actinomycosis of the jaw, and it seems therefore certain that many recover after simple incision of the abscess, and even through a natural rupture of it.

2. It is surprising to find, on microscopic examination of sections, how infrequently the colonies are found in the walls of the abscesses, though the pus contained many granules. The surrounding connective tissue probably proves an effective barrier to the spread of the disease.

Simple opening, curetting, and drainage have proved efficient in many cases; though recurrences may be frequent, healing eventually takes place. Where possible, excision of the inner half of the abscess-wall or sinus is the best treatment. The danger from swallowing the granules, where the discharge empties into the mouth, is hard to estimate. Certain cases of generalized disease, in the lungs, intestinal tract, liver, etc., occur in which the organism gained entrance through the food, or was swallowed, and therefore the surgeon should aim at making *external* drainage. This question is often a difficult one to decide. On the one hand, he wishes to avoid a scar on the face, especially in women; on the other, he wishes thoroughly to eradicate the disease; for with recurrence the scars would probably be worse than from a single, thorough, and clean operation. The individual case and the severity of the infection must determine the choice between curetting and cauterizing the cavity with tincture of iodine or carbolic acid and a more radical excision.

Iodide of potash, in doses of twenty grains three or four times a day, has distinctly influenced some cases for good, and should be used in connection with the local treatment.

Finally, I trust that when your attention has once been called to actinomycosis of the jaw, you will be enabled, through this brief paper, to make an early diagnosis of the disease, and I feel sure that a careful examination of chronic alveolar abscesses will show that the streptothrix actinomycotica is the cause of perhaps one-eighth or one-tenth of them.

